# Pathological features and molecular signatures of early olfactory dysfunction in 3xTg‐AD model mice

**DOI:** 10.1111/cns.14632

**Published:** 2024-02-17

**Authors:** Haitao Yu, Fangzhou Wang, Dongdong Jia, Shuguang Bi, Juan Gong, Jia‐Jun Wu, Yumin Mao, Jia Chen, Gao‐Shang Chai

**Affiliations:** ^1^ Department of Fundamental Medicine, Wuxi School of Medicine Jiangnan University Wuxi Jiangsu P. R. China; ^2^ Affiliated Hospital of Jiangnan University Wuxi Jiangsu Province P. R. China; ^3^ The Affiliated Mental Health Center of Jiangnan University, Wuxi Central Rehabilitation Hospital Wuxi Jiangsu P. R. China

**Keywords:** Alzheimer's disease (AD), FosB, neuroinflammation, neuronal activity, Nr4a2, olfactory dysfunction

## Abstract

**Background:**

Olfactory dysfunction is known to be an early manifestation of Alzheimer's disease (AD). However, the underlying mechanism, particularly the specific molecular events that occur during the early stages of olfactory disorders, remains unclear.

**Methods:**

In this study, we utilized transcriptomic sequencing, bioinformatics analysis, and biochemical detection to investigate the specific pathological and molecular characteristics of the olfactory bulb (OB) in 4‐month‐old male triple transgenic 3xTg‐AD mice (PS1M146V/APPSwe/TauP301L).

**Results:**

Initially, during the early stages of olfactory impairment, no significant learning and memory deficits were observed. Correspondingly, we observed significant accumulation of amyloid‐beta (Aβ) and Tau pathology specifically in the OB, but not in the hippocampus. In addition, significant axonal morphological defects were detected in the olfactory bulb, cortex, and hippocampal brain regions of 3xTg‐AD mice. Transcriptomic analysis revealed a significant increase in the expression of neuroinflammation‐related genes, accompanied by a significant decrease in neuronal activity‐related genes in the OB. Moreover, immunofluorescence and immunoblotting demonstrated an activation of glial cell biomarkers Iba1 and GFAP, along with a reduction in the expression levels of neuronal activity‐related molecules Nr4a2 and FosB, as well as olfaction‐related marker OMP.

**Conclusion:**

In sum, the early accumulation of Aβ and Tau pathology induces neuroinflammation, which subsequently leads to a decrease in neuronal activity within the OB, causing axonal transport deficits that contribute to olfactory disorders. Nr4a2 and FosB appear to be promising targets for intervention aimed at improving early olfactory impairment in AD.

## Graphical abstract

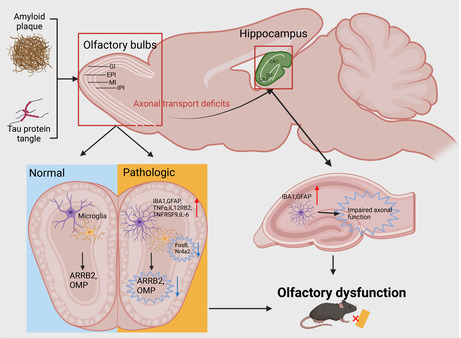

## 1 INTRODUCTION

Alzheimer's disease (AD) is the most prevalent neurodegenerative disease characterized by the accumulation of amyloid‐beta (Aβ) and Tau proteins, leading to cognitive decline and memory loss.^1^ However, even before the onset of cognitive impairment, individuals with AD commonly experience significant olfactory impairment, indicating that olfactory dysfunction is an early feature of the disease.^2^ Unfortunately, the challenge of obtaining brain tissue samples from individuals with early‐stage anosmia has hindered the identification of biomarkers associated with early olfactory impairment in AD. Nevertheless, studying AD model mice allows us to investigate the pathological and molecular characteristics of early olfactory disorders.^3^, ^4^, ^5^ This approach provides valuable insights into the relationship between olfactory dysfunction and the early diagnosis and treatment of AD.

Our previous studies have shown that olfactory dysfunction can be used as an early screening tool,^6^ but the specific mechanism remains unclear. It has been reported that neuropathological markers of AD, such as Aβ and Tau pathology, are present in areas associated with olfactory function, particularly the olfactory bulb (OB), in aging and at autopsy of different neurodegenerative diseases.^7^ OB is also believed to be an entry point for potential pathogens to enter and spread throughout the brain.^8^, ^9^ The presence of Tau and α‐synuclein (αSyn) histopathology in the OB has been reported in the early stages of AD, Lewy body disease (LBD), Parkinson's disease (PD), and multiple system atrophy (MSA).^10^, ^11^ Similar findings have been observed in other neurodegenerative diseases such as amyotrophic lateral sclerosis, progressive supranuclear palsy, corticobasal degeneration, Pick's disease, and Huntington's disease.^12^, ^13^, ^14^ These findings provide a potential model for understanding the pathogenesis of olfactory‐mediated neurodegenerative diseases, including AD and LBD. Furthermore, the presence of Aβ pathology in the OB may serve as an indicator of AD diagnosis. Studies have shown a correlation between Aβ in the OB and the overall amyloid phase and Braak neurofibrillary tangle phase in the brain.^7^, ^11^ Overall, these findings contribute to our understanding of the role of olfactory dysfunction in neurodegenerative diseases and highlight the potential for using olfactory markers in diagnostics.

Triple transgenic AD mice (3xTg‐AD) (Stock No: 34830, 129S4.CgTg [APPSwe, tauP301L] 1LfaPsen1tm1Mpm/Mmjax) began to exhibit learning and memory deficits at 6.5 months of age^15^ and olfactory dysfunction at 3–5 months of age.^3^ Here, we identified 4‐month‐old 3xTg‐AD mice with pronounced olfactory dysfunction, but without significant impairment in learning and memory, as a suitable model for investigating biomarkers of early olfactory dysfunction. Correspondingly, significant Aβ and Tau pathology was observed in the olfactory bulb (OB) but not in the hippocampus of 4‐month‐old 3xTg‐AD mice. However, significant axonal morphological defects were observed from olfactory bulb to hippocampus in 3xTg‐AD mice. Transcriptomic analysis revealed genes related to neuroinflammation were significantly activated, while genes related to neuronal activity were significantly decreased. Validation experiments confirmed the glial cell markers Iba1 and GFAP were significantly activated, while neuronal activity‐related molecules Nr4a2 and FosB were significantly decreased within the OB. Nr4a2 and FosB appear to be promising biomarkers and potential targets for intervention in cases of early olfactory dysfunction.

## 2 RESULTS

### 2.1 Olfactory impairment predates cognitive memory impairment in 3xTg‐AD mice

**FIGURE 1:**
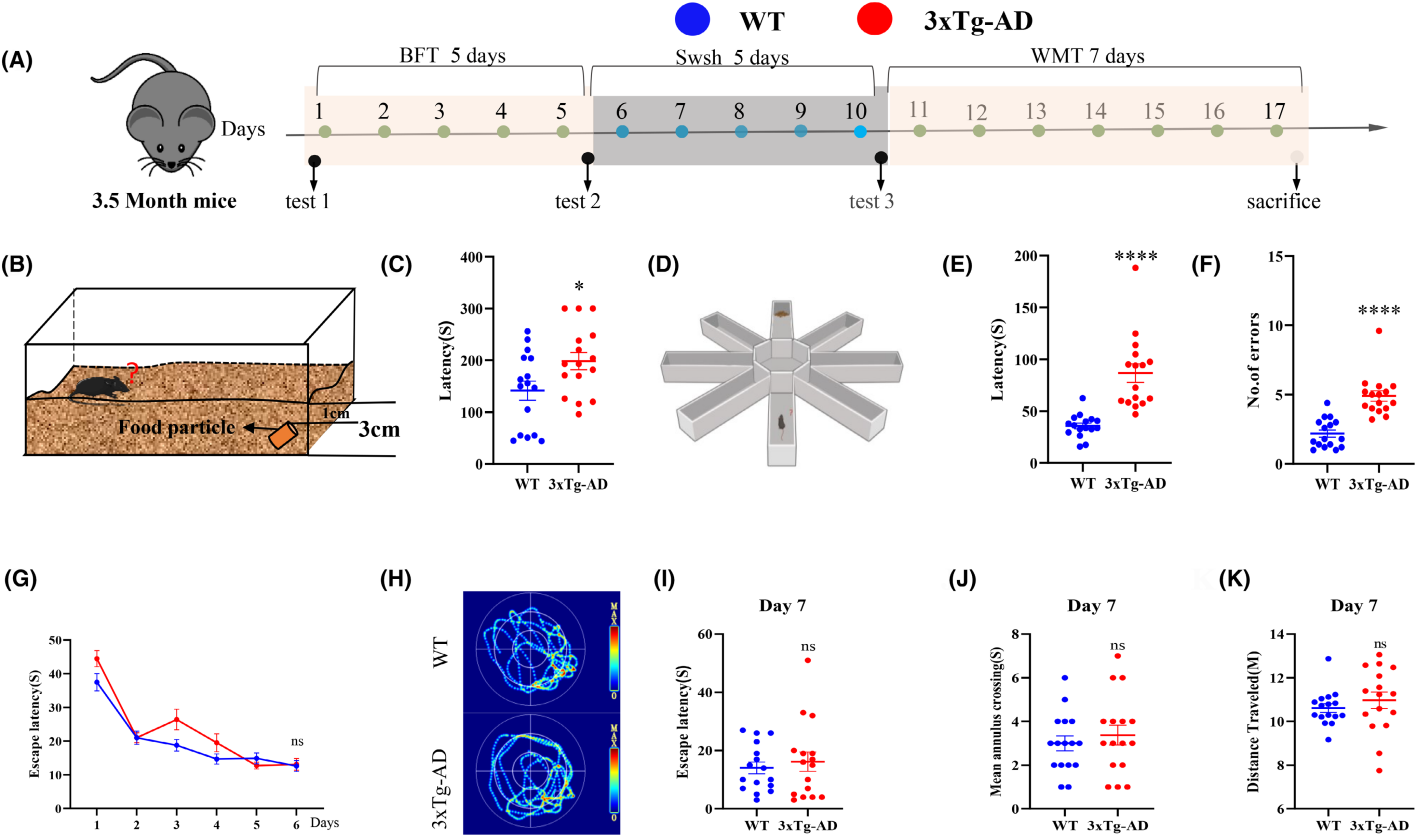
Olfactory impairment predates cognitive memory impairment in 3xTg‐AD mice. (A) Behavioral test workflow. (B,C) Buried food test showed that 3xTg‐AD mice took longer to find food. (D–F) The cookie‐finding test results showed that 3xTg‐AD entered the wrong arm more often and took more time to find food. (G) Escape latency to the hidden platform between Days 1 and 6. (H) Swimming pathway traveled to locate the platform on Day 7. (I) The escape latency, (J) number of crossings of the original position of the platform, (K) traveled distance on Day 7. Data were shown as mean ± SEM. *p < 0.05, **p < 0.01, ***p < 0.001; ns, not significant. *N* = 16 for each group.

In order to detect olfactory and learning and memory deficits in 3xTg‐AD mice at the age of 3–4 months, several behavioral tests were performed, such as buried food test, cookie‐finding test, and Morris water maze test (Figure 1). The buried food test showed that 3xTg‐AD mice had a longer latency to find the buried food than WT mice (Figure 1B,C). The cookie‐finding test showed that 3xTg‐AD mice entered the wrong arms more often than wild‐type mice and had a longer latency ratio to first enter the correct arm than wild‐type mice (Figure 1D–F). However, in the water maze test, it was observed that 4‐month‐old 3xTg‐AD mice did not display any learning impairment in comparison to wild‐type mice during the training phase (Figure 1G). Additionally, during the memory retrieval stage, there were no significant differences observed in terms of the latency to find the platform, the number of crossings over the platform, or the distance traveled (Figure 1H–K).

These findings indicate that 3xTg‐AD mice exhibit olfactory dysfunction while showing no significant impairments in learning and memory, which is in line with the development of behavioral disorders observed in patients, olfactory dysfunction occurs in the early stages of Alzheimer's disease (AD).^16^

### 2.2 Pathological changes of Tau and Aβ were observed in the olfactory bulb of 4‐month‐old 3xTg‐AD mice

**FIGURE 2:**
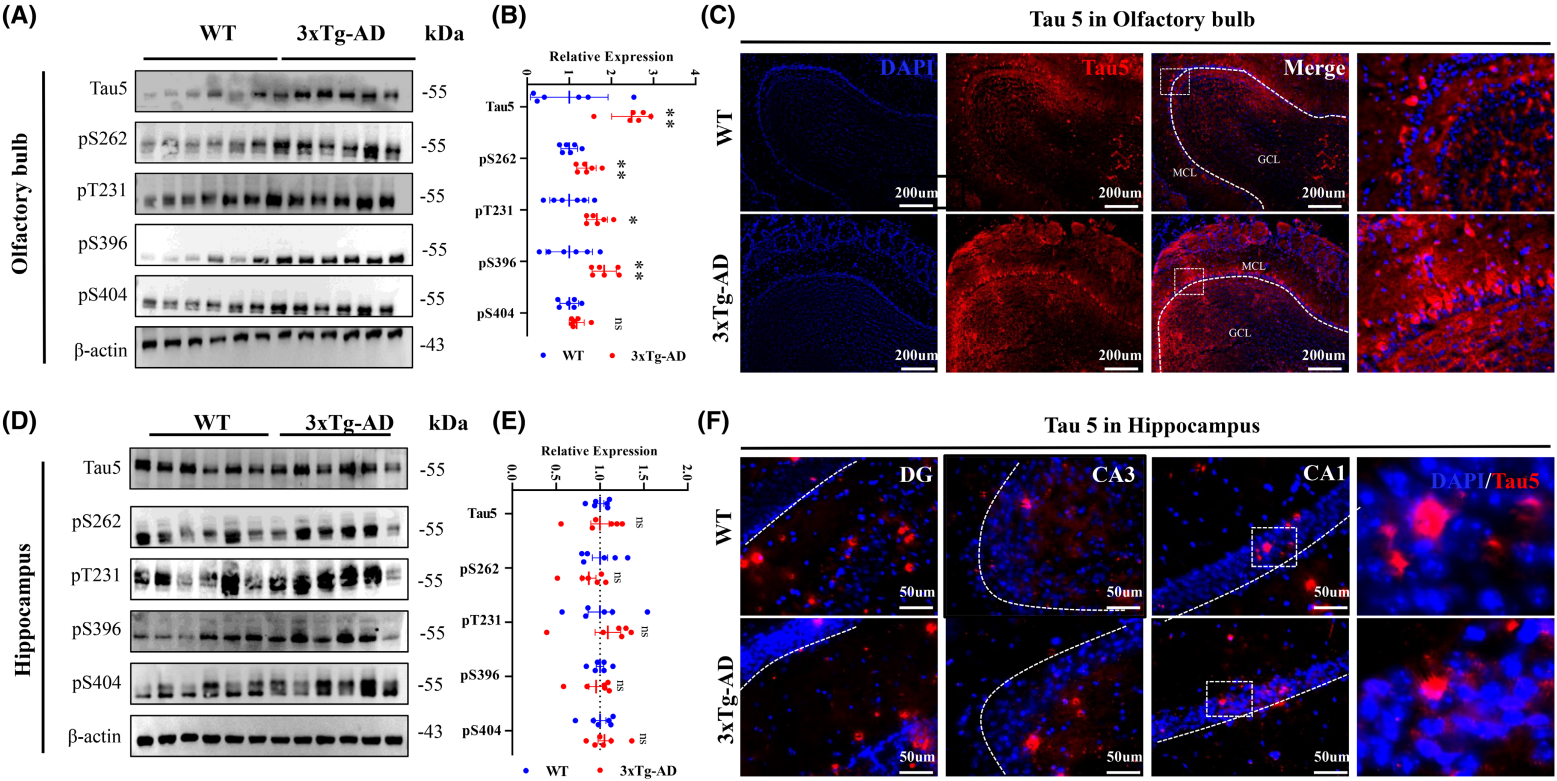
Pathological changes of Tau were observed in the olfactory bulb of 4‐month‐old 3xTg‐AD mice. (A,B,D,E) Hyperphosphorylated tau (pS262, pT231, pS396, Ps404) and total tau (Tau5) were increased in olfactory bulb (OB; A,B), while no significant changes were observed in the hippocampus (D,E) subset of 4‐month‐old 3xTg‐AD mice compared with age‐ and sex‐matched wild‐type mice measured by Western blotting. *N* = 6 for each group. (C,F) Expression of Tau pathology in the OB (C) and hippocampus (F) of 3xTg‐AD mice measured by immunofluorescence staining.

**FIGURE 3:**
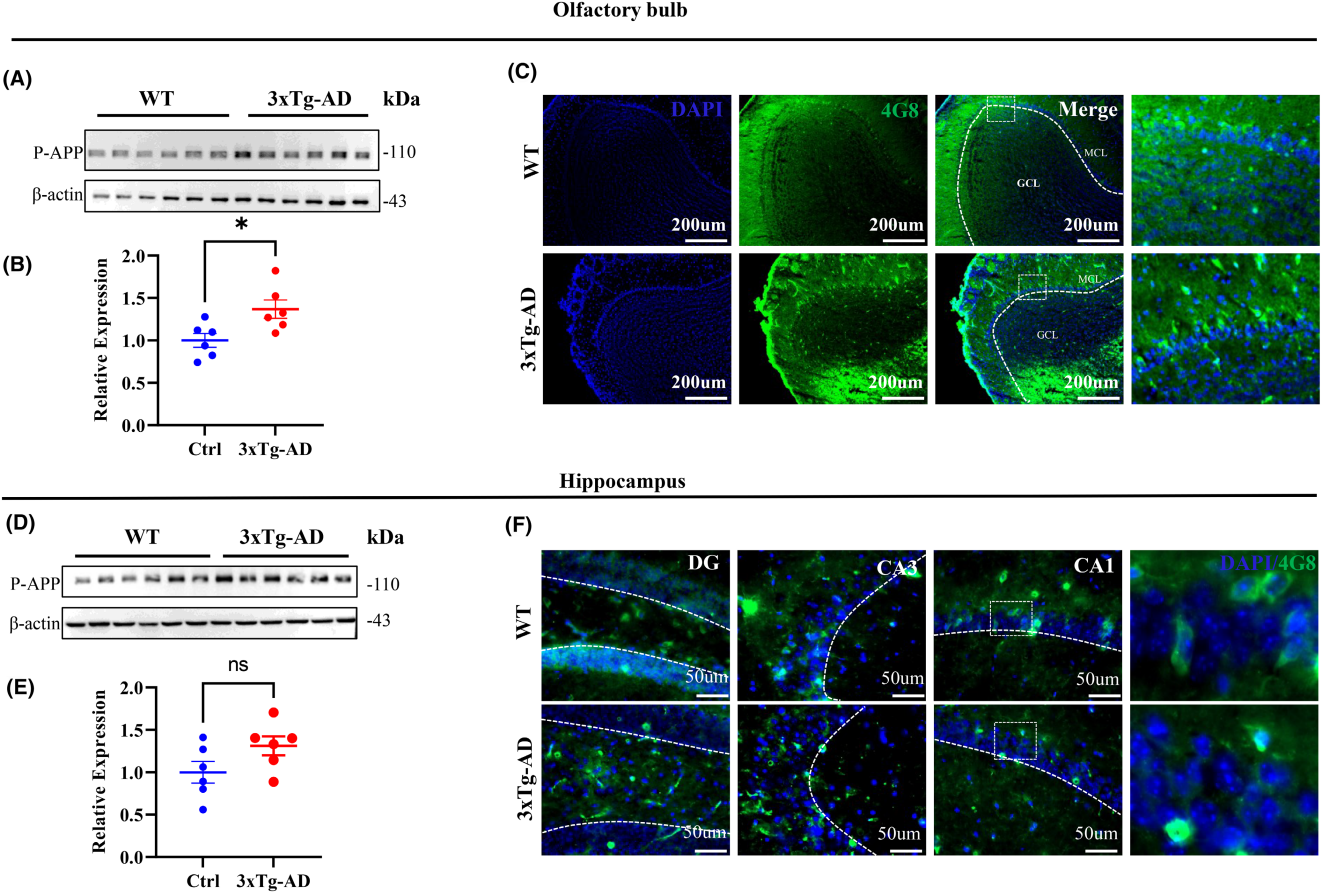
Pathological changes of Aβ‐related protein were observed in the olfactory bulb of 4‐month‐old 3xTg‐AD mice. (A,B,D,E) P‐APP were increased in olfactory bulb (OB; A,B), while no significant changes were observed in the hippocampus (D,E) subset of 4‐month‐old 3xTg‐AD mice compared with age‐ and sex‐matched wild‐type mice measured by Western blotting. *N* = 6 for each group. (C,F) Expression of Aβ pathology (4G8) in the OB (C) and hippocampus (F) of 3xTg‐AD mice measured by immunofluorescence staining.

In order to study the pathological changes of Tau and Aβ in the olfactory bulb (OB) of 3xTg‐AD mice, we conducted an examination of Tau protein levels and its phosphorylation sites, P‐APP. We discovered that Tau5, Ser262, Thr231, and Ser396 were significantly elevated in the OB (Figure 2). Additionally, we observed increased levels of P‐APP and 4G8 in the OB, but not in the hippocampus (Figure 3). These findings align with our behavioral measurements and provide evidence that increased Tau and Aβ pathology may contribute to olfactory dysfunction in 3xTg‐AD mice.

### 2.3 The significant axonal morphological defects were detected in the olfactory bulb, cortex, and hippocampal brain regions of 4‐month‐old 3xTg‐AD mice

**FIGURE 4:**
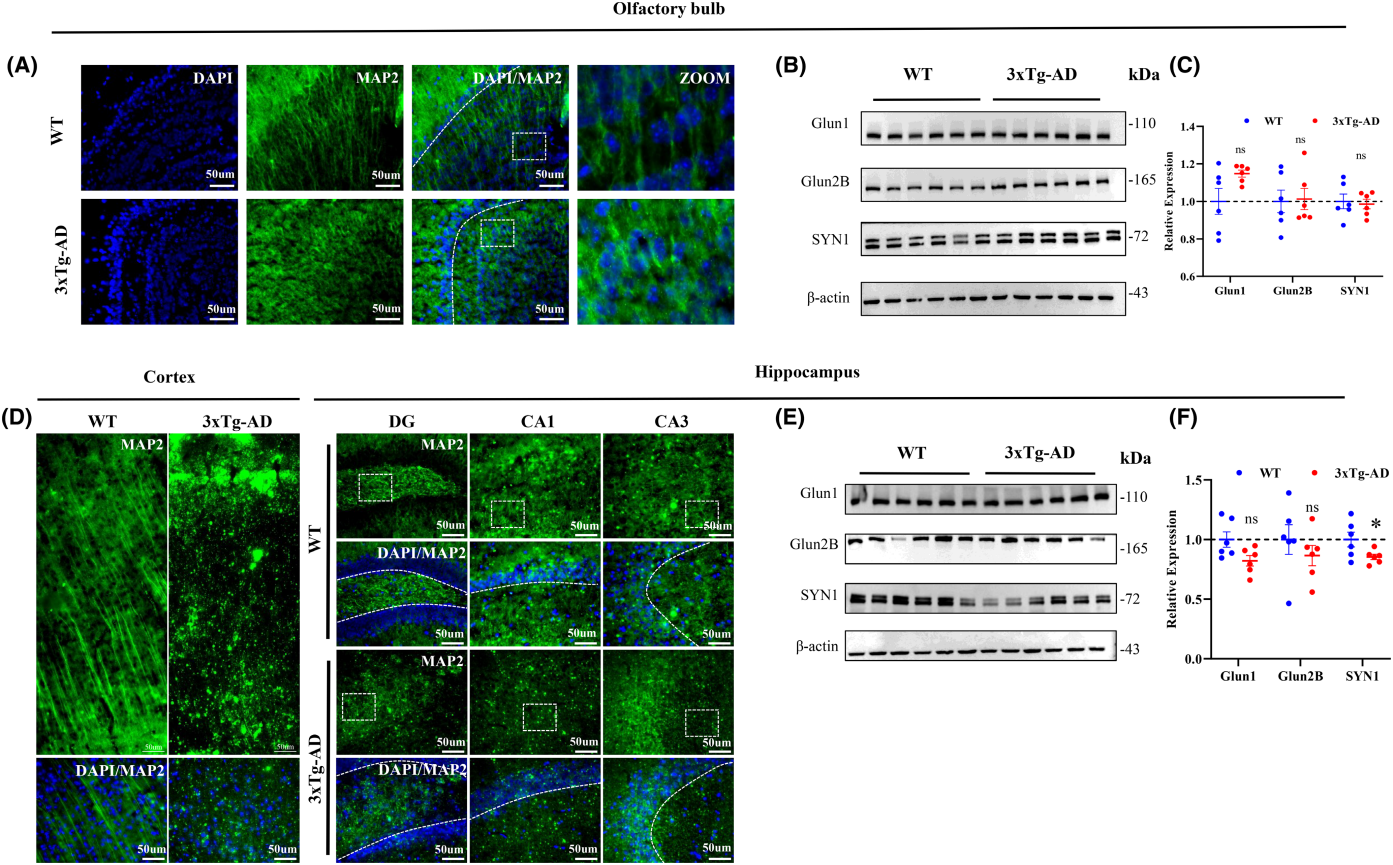
The significant axonal morphological defects were detected in the olfactory bulb, cortex, and hippocampal brain regions of 4‐month‐old 3xTg‐AD mice. (A,D) Expression of MAP2 in the OB (A) and cortex, hippocampus (D) of 3xTg‐AD mice measured by immunofluorescence staining. (B,C) The protein levels of GluN1, GluN2B, SYN1 in OB were tested by Western blotting (*n* = 6). (E,F) The protein levels of GluN1, GluN2B, SYN1 in hippocampus were tested by Western blotting (*n* = 6). Data were shown as mean ± SEM. *p < 0.05; ns, not significant.

Since axons and synapses are crucial for neural electrical signal transduction, we first examined microtubule‐associated protein 2 (MAP2) and found that MAP2 was significantly reduced in the olfactory bulb, cortex, and hippocampus of 3xTg‐AD mice, along with disrupted axonal morphology (Figure 4A,D). To evaluate synaptic function in mice, we examined synapse‐associated proteins (Glun1, Glun2B, and SYN1). Western blot showed that synapse‐associated proteins SYN1 showed a slight decrease in hippocampus, and other synapse‐associated proteins did not change significantly in OB and hippocampus (Figure 4B,C,E,F). These findings suggest that synapse‐associated protein expression remained relatively stable in the OB and hippocampus of the 4‐month‐old 3xTg‐AD mice. These results suggest that the destruction of axons may affect the transmission of olfactory signals through the OB to the hippocampus.

### 2.4 Transcriptome analysis revealed significant dysregulation of neuronal activity and immune inflammation‐related processes in the olfactory bulb of 3xTg‐AD mice

**FIGURE 5:**
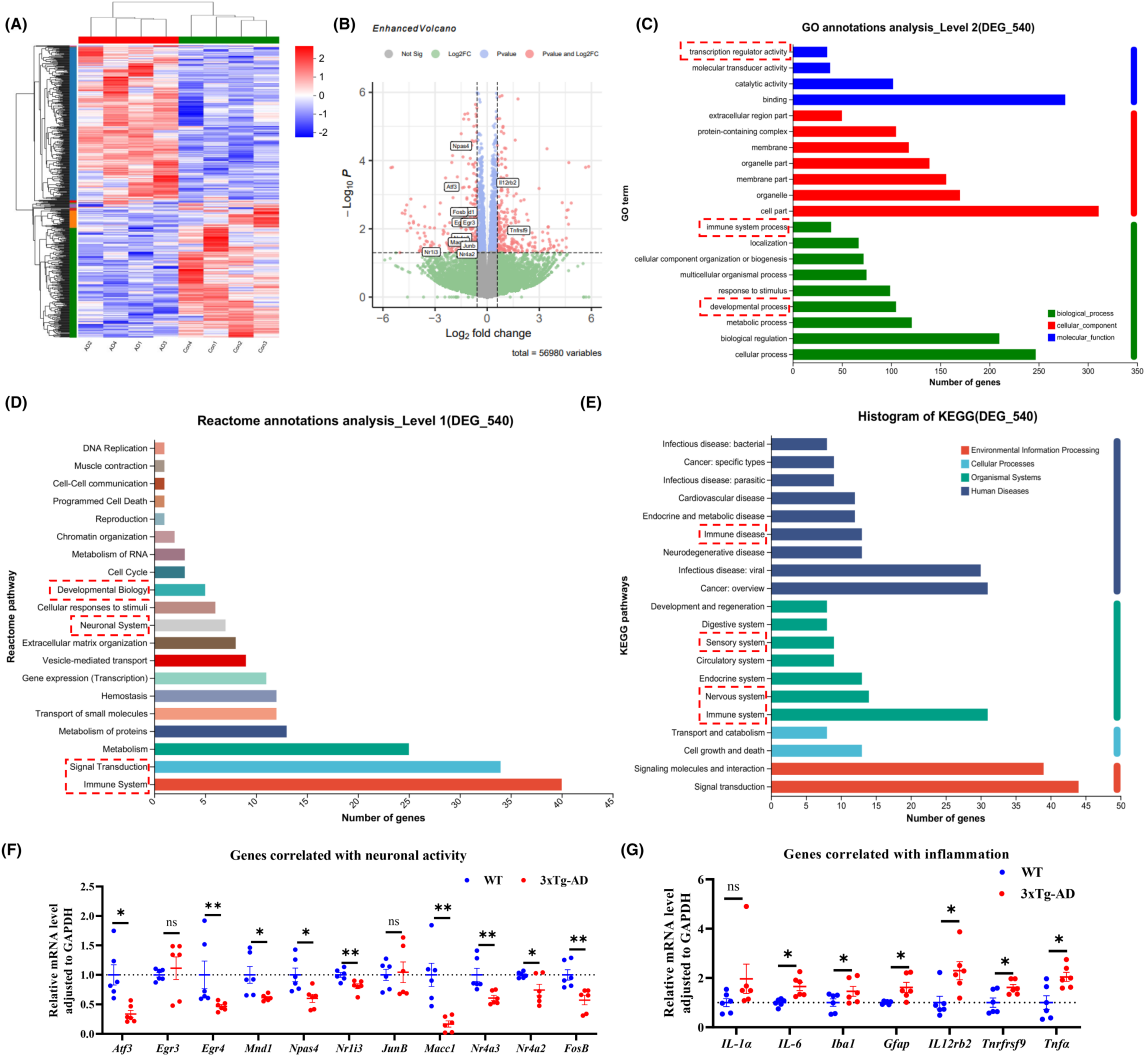
Transcriptome analysis revealed significant dysregulation of neuronal activity and immune inflammation‐related processes in the olfactory bulb of 3xTg‐AD mice. (A,B) The heatmap of differentially expressed genes (DEGs; *p* < 0.05, fold change >1.5) in the OB for 3xTg‐AD vs. WT mice. The *Z* value of gene abundance was plotted in a red‐blue color scale, with red and blue indicating increased and decreased protein expression, respectively (A). The red color of the volcano plot represents significantly differentially expressed genes (B). (C–E) Gene ontology (GO; C), reactome annotations (D) and Kyoto Encyclopedia of Genes and Genomes (KEGG; E) enrichment analysis of DEGs were performed. (F,G) Quantitative real‐time PCR (qRT‐PCR) verification of specific target genes in volcano plot, genes correlated with neuronal activity (F; *n* = 6 for each group), genes correlated with inflammation (G; *n* = 6 for each group). Data were shown as mean ± SEM. *p < 0.05, **p < 0.01; ns, not significant. *N* = 6 for each group.

Transcriptomics found 540 genes that were differentially expressed (DEGs) in the OB of the 3xTg‐AD mice, with 220 up‐regulated and 200 down‐regulated (Figure 5A,B). Gene ontology (GO) analysis showed that DEGs were mainly involved in immune system processes, transcription regulation, and developmental process (Figure 5C). Reactome annotations analysis revealed that DEGs were mainly enrichment in immune system, signal transduction, nervous system, developmental process, cell cycle, and DNA replication (Figure 5D). KEGG enrichment analysis showed that DEGs were mainly involved in signal transduction, nervous system, immune system, sensory system, and immune disease (Figure 5E). Overall, the analysis revealed changes in neuronal transcriptional activity regulation and immune inflammatory systems in the OB of the 3xTg‐AD mice.

Specifically, the DEGs involved in neuronal transcriptional activity mainly include Mnd1, Nr1i3, Macc1, Npas4, Atf3, Nr4a2, Junb, FosB, Egr3, Nr4a3, Egr4 and were all down‐regulated in the OB of 3xTg‐AD mice (Figure 5B). The DEGs related to immune inflammation mainly included Tnfrsf9 and Il12rb2, which were significantly up‐regulated in the OB of 3xTg‐AD mice (Figure 5B). Consistent with the transcriptional results, we confirmed the down‐regulation of key neuronal transcriptional activity factors and up‐regulation of inflammation‐related factors by qRT‐PCR. (Figure 5F). Additionally, we found that the transcription levels of neuroinflammation‐related genes IL‐6, TNF‐α, Iba1, and GFAP were significantly elevated in the OB of the 3xTg‐AD mice (Figure 5G). This provides further evidence of altered neuronal activity regulation and increased inflammatory response in the OB of these mice.

### 2.5 The expression of glial cell markers Iba1 and GFAP in brain tissue of 4‐month‐old 3xTg‐AD mice

**FIGURE 6:**
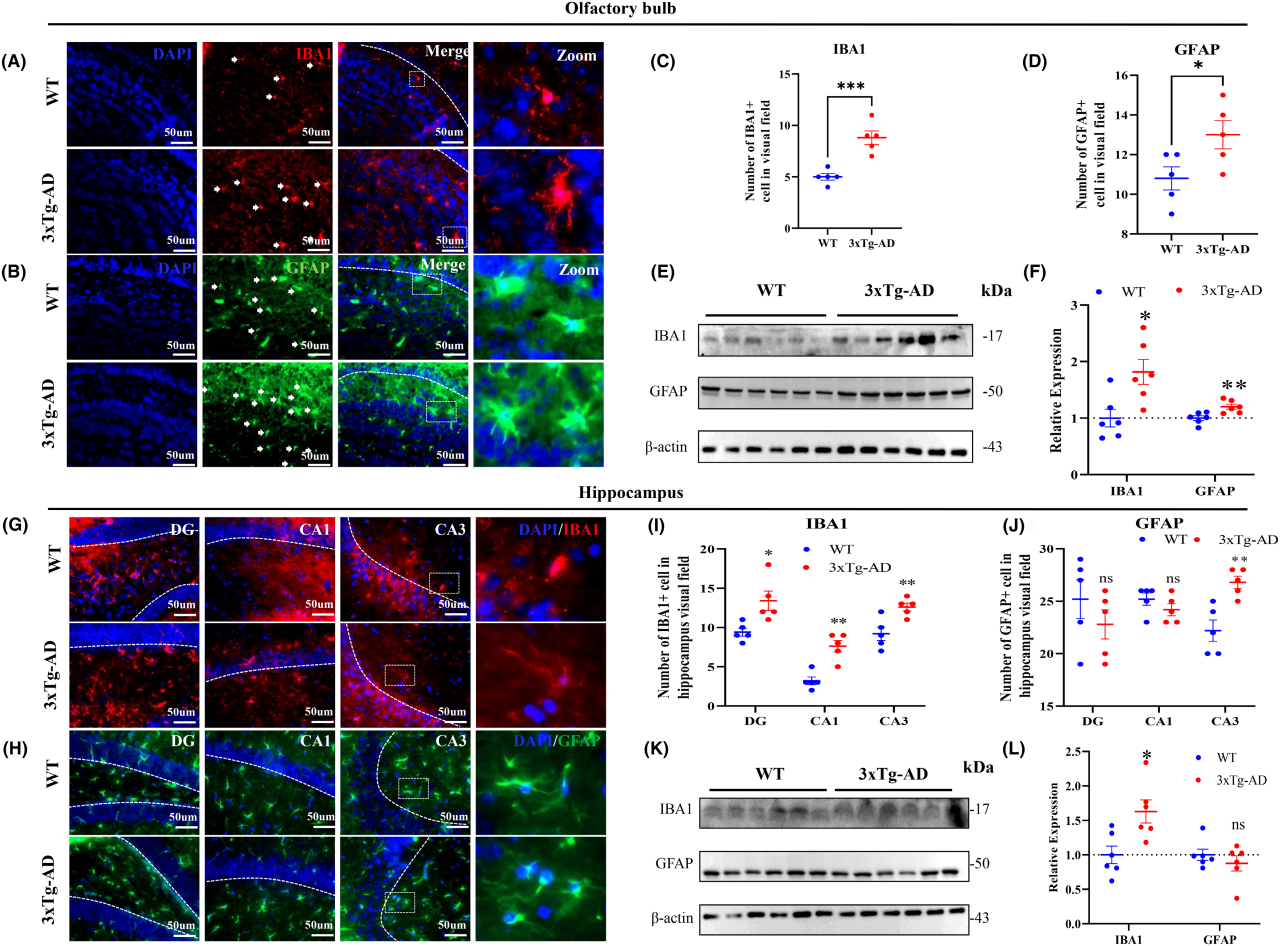
The expression of glial cell markers Iba1 and GFAP in brain tissue of 4‐month‐old 3xTg‐AD mice. (A–D) Representative immunofluorescence of Iba‐1 and GFAP in the OB (*n* = 5 for each group). (E,F) Expression of Iba1 and GFAP in the OB of 3xTg‐AD mice measured by Western blotting (*n* = 6 for each group). (G–J) Representative immunofluorescence of Iba‐1 and GFAP in the hippocampus (*n* = 5 for each group). (K,L) Expression of Iba1 and GFAP in the hippocampus of 3xTg‐AD mice measured by Western blotting (*n* = 6 for each group). Data were shown as mean ± SEM. *p < 0.05, **p < 0.01; ns, not significant.

The activation of glial cells, specifically astroglia and microglia, is closely associated with neuroinflammation. To assess this activation, we measured the levels of the astrocyte marker GFAP and the microglia marker Iba1 using Western blotting and immunofluorescence techniques. The results demonstrated significant activation of both GFAP and Iba1 in the olfactory bulb of 4‐month‐old 3xTg‐AD mice (Figure 6A–F). Interestingly, in the 3xTg‐AD hippocampus, there was a significant increase in Iba1 expression, while GFAP expression was no significantly changed, and significant activation was observed only in the CA3 region (Figure 6G–L). These findings indicate glial cells activation in both the olfactory bulb and hippocampus of 4‐month‐old 3xTg‐AD mice.

### 2.6 The expression of neuronal activity‐related molecules Nr4a2 and FosB in the OB of 4‐month‐old 3xTg‐AD mice

**FIGURE 7:**
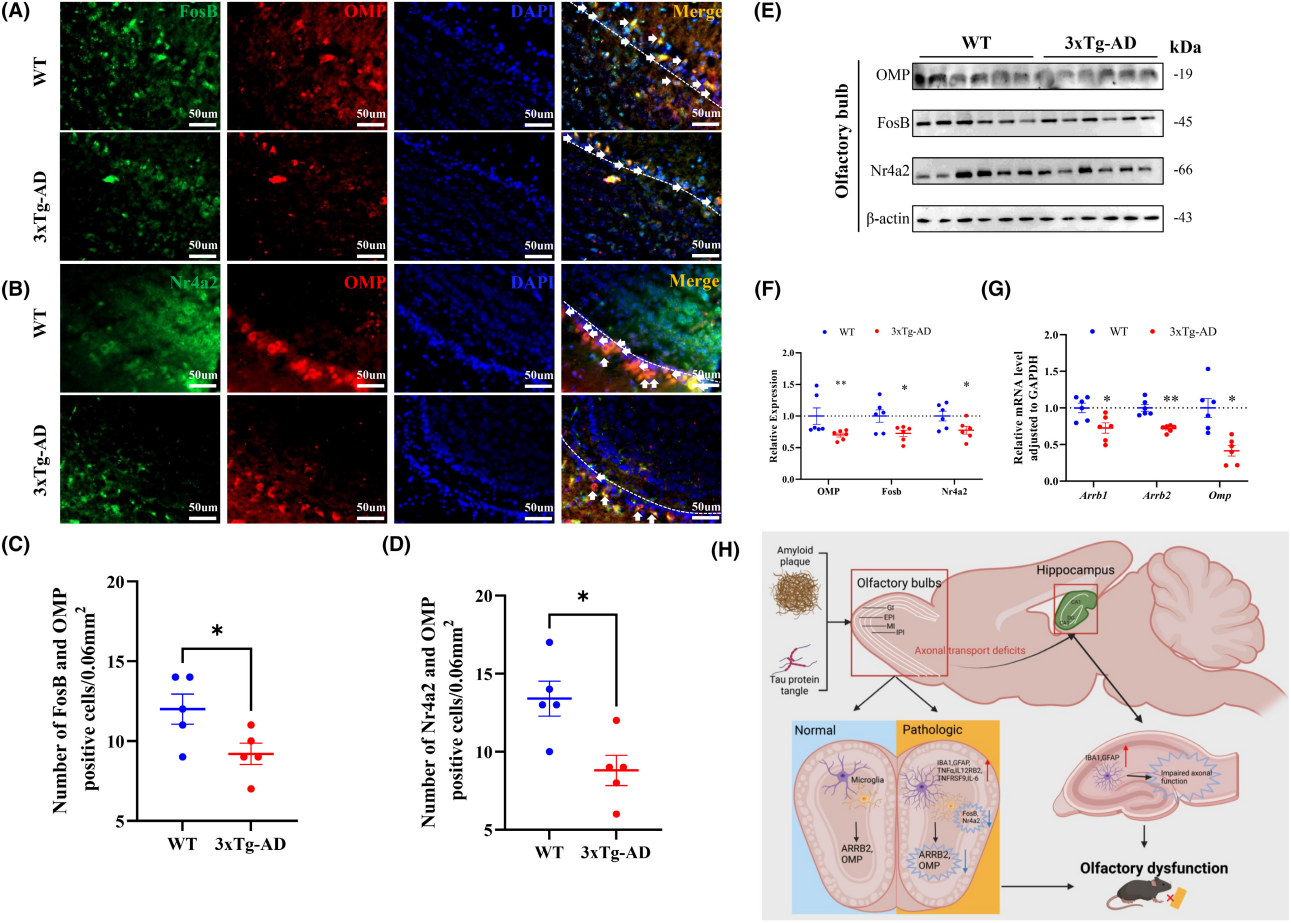
The expression of neuronal activity‐related molecules Nr4a2 and FosB in the OB of 4‐month‐old 3xTg‐AD mice. (A,C) Immunofluorescence staining of FosB+ and OMP+ cells in OB. Scale bar, 50 μm (A); statistic results of FosB+ and OMP+ cells numbers in OB (C; *n* = 5 for each group). (B,D) Immunofluorescence staining of Nr4a2+ and OMP+ cells in OB. Scale bar, 50 μm (B); statistic results of Nr4a2+ and OMP+ cells numbers in OB (D; *n* = 5 for each group). (E,F) Expression of OMP, FosB, Nr4a2 in the OB of 3xTg‐AD mice measured by Western blotting (*n* = 6 for each group). (G) Quantitative real‐time PCR (qRT‐PCR) verification of Arrb1, Arrb2 and OMP, genes correlated with neuronal Signaling (G; *n* = 6 for each group). (H) Diagram of the early accumulation of Aβ and Tau pathology induces neuroinflammation, which subsequently leads to a decrease in neuronal activity within the OB, causing axonal transport deficits that contribute to olfactory disorders. Data were shown as mean ± SEM. *p < 0.05, **p < 0.01.

Nr4a2 and FosB are transcription factors known to have essential roles in the regulation of neuronal activity.^17^ Specifically, chronic inflammation, induced by lipopolysaccharide (LPS) stimulation, has been associated with damage to neuronal and synaptic functions. Activation of Nr4a2 has demonstrated protective effects against the neurotoxic effects caused by chronic inflammation, potentially due to its ability to mitigate damage to neuronal and synaptic functions. Using immunoblotting and immunofluorescence experiments, we observed a significant reduction in the expression of Nr4a2 and FosB, as well as a decrease in colocalization with the olfactory biomarker OMP (Figure 7A–F). Overall, our findings suggest that the dysregulation of Nr4a2 and FosB may lead to impaired neuronal activity in the olfactory system, potentially resulting in olfactory dysfunction.

Additionally, odor receptors represent the largest subfamily of G protein‐coupled receptors and play a vital role in olfactory signal transduction. β‐arrestin1 (Arrb1) and β‐arrestin2 (Arrb2) not only regulate G protein‐coupled receptors (GPCRs) endocytosis and desensitization but also contribute to the initiation of alternative signaling pathways, thereby participating in the regulation of olfactory function.^18^, ^19^ Our data showed a significant decrease in the mRNA levels of Arrb1 and Arrb2, which could potentially impact olfactory signaling by regulating GPCRs (Figure 7G).

Overall, this study deepens our understanding of the molecular mechanisms underlying olfactory dysfunction and highlights the importance of Nr4a2, FosB, Arrb1, and Arrb2 in maintaining normal neuronal activity and olfactory function (Figure 7H). Further research is needed to elucidate the specific signaling pathways involved and to explore therapeutic strategies targeted at these transcription factors and their associated signaling molecules to potentially restore olfactory function in cases of dysfunction.

## 3 DISCUSSION

Olfactory dysfunction is an early symptom of Alzheimer's disease (AD) that occurs before cognitive impairment.^20^, ^21^ However, the current understanding of this dysfunction in AD patients is limited, which hampers the development of biomarkers and targeted drugs for early olfactory dysfunction in AD. This study found that 4‐month‐old 3xTg‐AD mice showed significant olfactory dysfunction despite no impairment in learning and memory function. Additionally, Tau and Aβ pathologies were observed in the 4‐month‐old 3xTg‐AD mice olfactory bulb (OB), although no significant changes were found in the hippocampus. This suggests that 4‐month‐old 3xTg‐AD mice may serve as an ideal animal model for studying the molecular markers of olfactory dysfunction in the early stages of AD. However, significant axonal morphological defects were observed from olfactory bulb to hippocampus in 3xTg‐AD mice. Further experiments demonstrated that early Tau and Aβ pathology may trigger inflammation in the brain, leading to reduced expression of Nr4a2 and FosB, resulting in decreased neuronal activity and olfactory disorders (Figure 7H). Therefore, Nr4a2 and FosB may be ultra‐early biomarkers and ideal therapeutic targets for olfactory dysfunction.

AD is the most prevalent degenerative disease among the elderly, and its main clinical manifestation is a progressive decline in learning and memory abilities.^1^ However, one of the earliest signs of the disease is olfactory dysfunction.^20^, ^21^ The sense of smell, which includes olfactory threshold, odor identification, and odor discrimination, is vital for mammals, and detecting olfactory dysfunction can be used as an early diagnostic tool for AD.^6^ The challenge lies in the fact that we can only analyze brain tissue post‐mortem, using cognitive scores and pathological grades, to determine the different stages of AD. Unfortunately, olfactory dysfunction occurs earlier in the disease progression, so we lack corresponding brain tissue samples to understand the underlying molecular events related to this dysfunction. This study suggested that 4‐month‐old 3xTg‐AD mice exhibit impaired sense of smell but no cognitive impairment. Moreover, the characteristic pathological features observed in AD, such as the clustering of Tau and Aβ pathology in the olfactory bulb, were also present in 4‐month‐old 3xTg‐AD mice at this early stage, while the hippocampus did not show obvious symptoms. This finding aligns with the progression of pathology observed in the brains of human AD patients. Therefore, these 4‐month‐old 3xTg‐AD mice may serve as an ideal model for investigating the molecular mechanisms underlying olfactory dysfunction during the early stages of AD.

Transcriptome analysis combined with bioinformatics system revealed the molecular characteristics of olfactory bulb in 4‐month‐old 3xTg‐AD mice and found that DEGs were mainly enriched in immune inflammatory system, neuronal transcriptional activity, and nervous system. AD is a chronic inflammatory disease, and pathways associated with Aβ and Tau production and aggregation as well as inflammation may converge and coordinate the progression of this neurodegenerative disease.^22^ Here, the production of Aβ and Tau may contribute to chronic inflammatory effects in the olfactory bulb region, which in turn leads to decreased neuronal activity and impaired olfactory signaling.

Olfactory bulb (OB) is an early affected brain region in AD patients, which has significant inflammatory effects and plays an important role in the onset and progression of AD.^23^, ^24^ Iba1 and GFAP are biomarkers of glial cells, and their increased expression indicates the activation of microglia and astrocytes.^25^ These glial cells are closely related to neuroinflammation, and their levels are significantly increased in multiple brain regions of patients with Alzheimer's disease (AD), including the olfactory bulb.^23^ In our study, we confirmed that Iba1 expression was increased in the olfactory bulb and hippocampus, GFAP expression was increased in the olfactory bulb of 4‐month‐old 3xTg‐AD mice, indicating significant glial activation and neuroinflammation.

Arrb1 and Arrb2, members of arrestin/beta‐arrestin protein family, are thought to participate in agonist‐mediated desensitization of G‐protein‐coupled receptors and cause specific dampening of cellular responses to stimuli such as hormones, neurotransmitters, or sensory signals. During the olfactory function, Arrb2 can ameliorate the neuroinflammation, which is considered to be the main factor causing inflammatory response. Previous studies have showed that the expression of Arrb1 and Arrb2 was mutually regulated in mouse models of PD and may have the opposite functions.^26^ Taken together, Arrb2 plays an important role in neural signal transduction and may affect olfactory function.

Nr4a2 is a transcription factor that plays a crucial role in neural development and is expressed in various regions of the central nervous system.^27^, ^28^ In AD, knocking down Nr4a2 has been found to significantly worsen the pathological symptoms of AD, while activation of Nr4a2 rescued age‐related memory decline and reduced neuroinflammation in the brain of mice.^29^, ^30^ Nr4a2 reduction has also been implicated in depression, where it mediates the decrease in neuronal activity induced by lipopolysaccharide (LPS), leading to the development of depressive symptoms.^17^ Furthermore, Nr4a2 is associated with age‐related macular degeneration,^31^ attention deficit hyperactivity disorder,^32^ cardiovascular abnormalities,^33^ and neuroinflammation in the brains of patients with Parkinson's disease (PD).^34^ FosB is indeed a member of the Fos family of transcription factors,^35^ which is part of the AP‐1 transcription factor complex and are involved in regulating gene expression.^36^ FosB is specifically considered a marker of chronic neuronal activation,^37^ while a decrease in its expression can indicate low neuronal activity.^17^ Here, we found that Nr4a2 and FosB are significantly reduced in the olfactory bulb and also in colocalization with OMP, suggesting that Nr4a2 and FosB may contribute to early olfactory dysfunction in AD via affecting neuronal activity.

## 4 CONCLUSIONS

In this study, we found that 4‐month‐old 3xTg‐AD mice can serve as a suitable model to study the molecular signals associated with ultra‐early olfactory dysfunction of AD. Significant Aβ and Tau pathology was observed in the olfactory bulb (OB) of 4‐month‐old 3xTg‐AD mice, along with the activation of genes associated with neuroinflammation and the reduction of genes related to neuronal activity, causing axonal transport deficits that contribute to olfactory disorders (Figure 7H). Nr4a2 and FosB are suggested as potential biomarkers for early olfactory dysfunction and may serve as targets for potential interventions.

## 5 MATERIALS AND METHODS

### 5.1 Experimental animals and sample preparation

Triple transgenic AD male mice (3xTg‐AD) (Stock No: 34830, 129S4.CgTg [APPSwe, tauP301L] 1LfaPsen1tm1Mpm/Mmjax) and wild‐type mice were a gift from Prof. Xifei Yang (Shenzhen Center for Disease Control and Prevention). The experimentation was authorized by the Animal Ethics Committee of Jiangnan University (JN. No20230830m0201215[349]). The area in which they were fed maintained a temperature range of 22–26°C, while the relative humidity was kept between 50%–60%, and adequate food and water were ensured, with 12‐h light–dark alternating. It is important to note that all experiments conducted with these mice adhere to the ethical considerations and animals' well‐being.^12^

### 5.2 Cookie‐finding test

As in the previous study,^3^ mice were given the opportunity to freely explore an eight‐arm maze for 5 min/day for a period of five days. After the acclimation period, the mice underwent five days of biscuit seeking trials. These trials were conducted once a day, with the biscuit placed at one end of the arm. The location of the biscuit was changed on a daily basis, ensuring that the mice did not rely on spatial memory to find the biscuit, but rather used their sense of smell. To evaluate the olfactory function of the mice, the number of entries into the wrong arm was recorded daily.

### 5.3 Buried food test

The food burial experiments conducted in this study followed the same procedures as described in previous studies,^3^, ^38^ which involved assessing odor familiarity, food deprivation, and detection on a daily basis. During the experiments, a bedding depth of 3 cm was used, and the food (a 10 mm peanut chocolate square) was buried beneath 1 cm in one of the corners of the cage. The time it took for the mice to find the food, known as the latency, was recorded. If the mice were unable to find the food within 5 min, a latency score of 300 s was recorded, and the test was stopped.

### 5.4 Morris water maze test

First, a series of learning and training experiments were conducted over six consecutive days. Mice were placed in the water maze from four quadrants and the time it took them to find the underwater platform was recorded. Memory detection experiments were performed on the second day after the learning and training phase. Several measures were recorded during this experiment, including the time it took for the mice to first cross the platform, the number of crossings made within 60 s, and the distance and time taken to reach the target quadrant. These measures allowed for an assessment of memory retention and spatial learning abilities in the mice.

### 5.5 Reverse transcription and real‐time quantitative PCR

Total RNA was extracted from mouse brain tissue by TRIzol method and reverse transcribed to produce complementary DNA (Vazyme Biotech Co., Ltd., China). Real‐time PCR was performed using 0.8 μL forward and 0.8 μL reverse primers, 1 μL cDNA, 10 μL SYBR Green PCR parent (Yeasen Biotechnology Co., Ltd., China), and 7.4 μL DEPC water. All the PCR primers employed are listed in Table 1.

**TABLE 1:** PCR primers employed in the present study.

Gene	Forward primer (5′ → 3′)	Reverse primer (5′ → 3′)
Atf3	CCAGGTCTCTGCCTCAGAAG	CATCTCCAGGGGTCTGTTGT
Egr3	AGACGTGGAGGCCATGTATC	GGGAAAAGATTGCTGTCCAA
Egr4	CTCCACCTGAGCGACTTCTC	TCCAGGAAGCAGGAGTCTGT
Mnd1	GTGGGGAGGAAAAGAGAACC	GTTCAGAGCCTCCAACTTGC
Npas4	TCATGAGTCTTGCCTGCATC	CACCATAGAGTGGCCCAGAT
Nr1i3	GGAGGACCAGATCTCCCTTC	GTGGAGGATCGACTCCAAAA
JunB	ATGTGCACGAAAATGGAACA	CCTGACCCGAAAAGTAGCTG
Macc1	TGAACTGATTGTGGCTCTGC	AGGCAGGTTTCCACATCATC
Nr4a3	TCAGCCTTTTTGGAGCTGTT	TGAAGTCGATGCAGGACAAG
Nr4a2	CGGTTTCAGAAGTGCCTAGC	TTGCCTGGAACCTGGAATAG
FosB	GAGGGAGCTGACAGATCGAC	TTCCTTAGCGGATGTTGACC
IL‐1β	GACCTTCCAGGATGAGGACA	AGGCCACAGGTATTTTGTCG
IL‐6	CCGGAGAGGAGACTTCACAG	TCCACGATTTCCCAGAGAAC
Iba1	CCGAGGAGACGTTCAGCTAC	GACCAGTTGGCCTCTTGTGT
Gfap	AGAAAACCGCATCACCATTC	TCACATCACCACGTCCTTGT
Il12rb2	AGTCACCAACCTGTCCCTTG	GAACAGGCCACAGTTCCATT
Tnrfrsf9	TGGTGAGCTTCTCTCCCAGT	ATCGGCAGCTACAAGCATCT
Tnfa	CCGATGGGTTGTACCTTGTC	CGGACTCCGCAAAGTCTAAG
Arrb1	GGCTACAGGAGCGACTCATC	TAGCCGCACAGAGTTCCTTT
Arrb2	AAGTCGAGCCCTAACTGCAA	GGTGAGGGTCACGAACACTT
Omp	GAAGCAGGATGGTGAGAAGC	GTCCAGAACCACGTTCCAGT

### 5.6 Transcriptome and bioinformatics analysis

Transcriptomics comprehensively characterized the olfactory bulb gene expression of 3xTg‐AD mice, including RNA purification, reverse transcription, library construction, and sequencing. To gain insights into the biological functions and pathways associated with the differentially expressed genes, the Kyoto Encyclopedia of Genes and Genomes (KEGG) and the WEB‐based GEne SeT AnaLysis Toolkit (http://www.webgestalt.org) were utilized for performing these analyses.

### 5.7 Western blotting assays

The tissues were lysed in RIPA buffer containing protease inhibitor mixture, sonicated and then dissolved on ice for 30 min (ultrasound parameters: 5 s each time, 7 times in total). Then the lysates were centrifuged at 16,000*g* for 15 min at 4°C, after taking the supernatant, add equal volume of 2× protein loading buffer for subsequent treatment. All the primary antibodies (Table 2) were used to detect Tau pathology (Tau5, pS262, pT231, pS396, pS404), Aβ pathology (P‐APP), synaptic plasticity (Glun1, Glun2B, SYN1), Glial cell markers (Iba1, GFAP), neuronal activity (FosB, Nr4a2), and olfaction‐related marker (OMP), and HRP‐linked secondary antibody (anti‐rabbit or anti‐mouse IgG conjugated to horseradish peroxidase, 1:5000).

**TABLE 2:** Antibodies used in the Western blotting analysis and their properties.

Antibody	Specificity	Type	Dilution for WB	Source	Catalogue number
β‐Actin	β‐Actin	Poly‐	1:5000	SAB	21,338
Tau5	Tau5	Mono‐	1:1000 (1:200 for IF)	SAB	21,570
pS262	Phospho‐Tau (Ser262)	Mono‐	1:1000	SAB	11,111
pT231	Phospho‐Tau (Thr231)	Mono‐	1:1000	SAB	21,099
pS396	Phospho‐Tau (Ser396)	Mono‐	1:1000	SAB	11,102
pS404	Phospho‐Tau (Ser404)	Mono‐	1:1000	Bioss	Bs‐2392R
P‐APP	P‐APP	Mono‐	1:1000	CST	6986
Glun1	NMDAR1	Mono‐	1:1000	SAB	49,488
Glun2B	NMDAR2B	Mono‐	1:1000	Abcam	ab183942
SYN1	Synapsin‐1	Poly‐	1:1000 (1:200 for IF)	Millipore	ab1543
MAP2	MAP2	Poly‐	1:200 for IF	SAB	32,723
IBA1	IBA1	Mono‐	1:1000 (1:200 for IF)	Proteintech	60,190‐1‐Ig
GFAP	GFAP	Mono‐	1:1000 (1:200 for IF)	Proteintech	66,827‐1‐Ig
OMP	Olfactory marker protein	Mono‐	1:1000 (1:200 for IF)	Abcam	ab183947
FosB	FosB	Mono‐	1:1000 (1:200 for IF)	Santa Cruz	Sc‐398,595
Nr4a2	Nurr1	Mono‐	1:1000 (1:200 for IF)	Santa Cruz	Sc‐376,984

### 5.8 Immunofluorescence

Brain sections were incubated with anti‐Tau5 primary antibody, anti‐4G8, anti‐Iba1, anti‐GFAP, anti‐FosB, anti‐Nr4a2, anti‐OMP primary antibody for 24 h at 4°C (Table 2). The second day, the brain sections were washed with 0.1% TritonX‐100 in PBS for 3 times and incubated with donkey‐anti‐rabbit Alexa Fluor 488 (Jackson ImmunoResearch, 711‐545‐152) or donkey‐anti‐mouse Alexa Fluor 594 (Jackson ImmunoResearch, 715‐585‐150) for 1 h at room temperature. Pictures were visualized by LSM710 (Zeiss Carl LSM 710, Germany).

### 5.9 Statistical analysis

Student's *t*‐test was used to evaluate the level of significance between the two groups with GraphPad Prism software 9 (GraphPad Software, Inc., La Jolla, CA, USA). All the data were tested for normality by “Shapiro–Wilk,” the data that do not conform to a normal distribution were subjected to a non‐parametric *t*‐test, while the data that conforms to a normal distribution were subjected to a parametric *t*‐test. The data were expressed as mean ± SEM. and *p* values <0.05 was considered to be significant.

## AUTHOR CONTRIBUTIONS

*Experimental design*: HY, DJ, GC. *Experimental methods*: HY, FW, JW, GC. *Data analysis*: HY, FW, JW, JG, YM, SB, JC. *Manuscript‐writing*: HY, DJ, GC.

## CONFLICT OF INTEREST STATEMENT

The authors declare that they have no conflict of interest to disclose.

## Data Availability

All data used to support the findings of this study are included within the article, and raw data are available from the corresponding author.

## References

[1] Scheltens P , De Strooper B , Kivipelto M , et al. Alzheimer's disease. Lancet. 2021;397(10284):1577‐1590. 33667416 10.1016/S0140-6736(20)32205-4 PMC8354300

[2] Bahar‐Fuchs A , Chetelat G , Villemagne VL , et al. Olfactory deficits and amyloid‐beta burden in Alzheimer's disease, mild cognitive impairment, and healthy aging: a PiB PET study. J Alzheimers Dis. 2010;22(4):1081‐1087. 20930316 10.3233/JAD-2010-100696

[3] Chen M , Chen Y , Huo Q , et al. Enhancing GABAergic signaling ameliorates aberrant gamma oscillations of olfactory bulb in AD mouse models. Mol Neurodegener. 2021;16(1):14. 33663578 10.1186/s13024-021-00434-7 PMC7934466

[4] Wu N , Rao X , Gao Y , Wang J , Xu F . Amyloid‐beta deposition and olfactory dysfunction in an Alzheimer's disease model. J Alzheimers Dis. 2013;37(4):699‐712. 23948910 10.3233/JAD-122443

[5] Cassano T , Romano A , Macheda T , et al. Olfactory memory is impaired in a triple transgenic model of Alzheimer disease. Behav Brain Res. 2011;224(2):408‐412. 21741995 10.1016/j.bbr.2011.06.029

[6] Gao Y , Liu Y , Zhang Y , et al. Olfactory threshold test as a quick screening tool for cognitive impairment: analysis of two independent cohorts. J Alzheimers Dis. 2023;93(1):169‐178. 36970911 10.3233/JAD-230023

[7] Attems J , Lintner F , Jellinger KA . Olfactory involvement in aging and Alzheimer's disease: an autopsy study. J Alzheimers Dis. 2005;7(2):149‐157; discussion 173–80. 15851853 10.3233/jad-2005-7208

[8] Rey NL , Wesson DW , Brundin P . The olfactory bulb as the entry site for prion‐like propagation in neurodegenerative diseases. Neurobiol Dis. 2018;109:226‐248. 28011307 10.1016/j.nbd.2016.12.013 PMC5972535

[9] Braak H , Rub U , Gai WP , Del Tredici K . Idiopathic Parkinson's disease: possible routes by which vulnerable neuronal types may be subject to neuroinvasion by an unknown pathogen. J Neural Transm (Vienna). 2003;110(5):517‐536. 12721813 10.1007/s00702-002-0808-2

[10] Beach TG , White CL 3rd , Hladik CL , et al. Arizona Parkinson's disease, olfactory bulb alpha‐synucleinopathy has high specificity and sensitivity for Lewy body disorders. Acta Neuropathol. 2009;117(2):169‐174. 18982334 10.1007/s00401-008-0450-7 PMC2631085

[11] Attems J , Walker L , Jellinger KA . Olfactory bulb involvement in neurodegenerative diseases. Acta Neuropathol. 2014;127(4):459‐475. 24554308 10.1007/s00401-014-1261-7

[12] Takeda T , Iijima M , Uchihara T , et al. TDP‐43 pathology progression along the olfactory pathway as a possible substrate for olfactory impairment in amyotrophic lateral sclerosis. J Neuropathol Exp Neurol. 2015;74(6):547‐556. 25933387 10.1097/NEN.0000000000000198

[13] Kovacs T , Papp MI , Cairns NJ , Khan MN , Lantos PL . Olfactory bulb in multiple system atrophy. Mov Disord. 2003;18(8):938‐942. 12889086 10.1002/mds.10466

[14] Yoshimura N . Olfactory bulb involvement in Pick's disease. Acta Neuropathol. 1988;77(2):202‐205. 3227817 10.1007/BF00687432

[15] Stover KR , Campbell MA , Van Winssen CM , Brown RE . Early detection of cognitive deficits in the 3xTg‐AD mouse model of Alzheimer's disease. Behav Brain Res. 2015;289:29‐38. 25896362 10.1016/j.bbr.2015.04.012

[16] Sohrabi HR , Bates KA , Weinborn MG , et al. Olfactory discrimination predicts cognitive decline among community‐dwelling older adults. Transl Psychiatry. 2012;2(5):e118. 22832962 10.1038/tp.2012.43 PMC3365262

[17] He Y , Wang Y , Yu H , et al. Protective effect of Nr4a2 (Nurr1) against LPS‐induced depressive‐like behaviors via regulating activity of microglia and CamkII neurons in anterior cingulate cortex. Pharmacol Res. 2023;191:106717. 36948326 10.1016/j.phrs.2023.106717

[18] Mashukova A , Spehr M , Hatt H , Neuhaus EM . Beta‐arrestin2‐mediated internalization of mammalian odorant receptors. J Neurosci. 2006;26(39):9902‐9912. 17005854 10.1523/JNEUROSCI.2897-06.2006 PMC6674477

[19] Merritt DM , MacKay‐Clackett I , Almeida SMT , Tran C , Ansar S , van der Kooy D . Arrestin‐mediated desensitization enables intraneuronal olfactory discrimination in *Caenorhabditis elegans* . Proc Natl Acad Sci USA. 2022;119(31):e2116957119. 35878038 10.1073/pnas.2116957119 PMC9351366

[20] Bathini P , Mottas A , Jaquet M , Brai E , Alberi L . Progressive signaling changes in the olfactory nerve of patients with Alzheimer's disease. Neurobiol Aging. 2019;76:80‐95. 30708185 10.1016/j.neurobiolaging.2018.12.006

[21] Li W , Howard JD , Gottfried JA . Disruption of odour quality coding in piriform cortex mediates olfactory deficits in Alzheimer's disease. Brain. 2010;133(9):2714‐2726. 20724290 10.1093/brain/awq209 PMC2948816

[22] Webers A , Heneka MT , Gleeson PA . The role of innate immune responses and neuroinflammation in amyloid accumulation and progression of Alzheimer's disease. Immunol Cell Biol. 2020;98(1):28‐41. 31654430 10.1111/imcb.12301

[23] Kohl Z , Schlachetzki JC , Feldewerth J , et al. Distinct pattern of microgliosis in the olfactory bulb of neurodegenerative Proteinopathies. Neural Plast. 2017;2017:3851262. 28409032 10.1155/2017/3851262 PMC5376461

[24] Doorn KJ , Goudriaan A , Blits‐Huizinga C , et al. Increased amoeboid microglial density in the olfactory bulb of Parkinson's and Alzheimer's patients. Brain Pathol. 2014;24(2):152‐165. 24033473 10.1111/bpa.12088 PMC8029318

[25] Norden DM , Trojanowski PJ , Villanueva E , Navarro E , Godbout JP . Sequential activation of microglia and astrocyte cytokine expression precedes increased Iba‐1 or GFAP immunoreactivity following systemic immune challenge. Glia. 2016;64(2):300‐316. 26470014 10.1002/glia.22930 PMC4707977

[26] Fang Y , Jiang Q , Li S , et al. Opposing functions of β‐arrestin 1 and 2 in Parkinson's disease via microglia inflammation and Nprl3. Cell Death Differ. 2021;28(6):1822‐1836. 33686256 10.1038/s41418-020-00704-9 PMC8184754

[27] Jeon SG , Yoo A , Chun DW , et al. The critical role of Nurr1 as a mediator and therapeutic target in Alzheimer's disease‐related pathogenesis. Aging Dis. 2020;11(3):705‐724. 32489714 10.14336/AD.2019.0718 PMC7220289

[28] Decressac M , Volakakis N , Björklund A , Perlmann T . NURR1 in Parkinson disease—from pathogenesis to therapeutic potential. Nat Rev Neurol. 2013;9(11):629‐636. 24126627 10.1038/nrneurol.2013.209

[29] Chatterjee S , Walsh EN , Yan AL , Giese KP , Safe S , Abel T . Pharmacological activation of Nr4a rescues age‐associated memory decline. Neurobiol Aging. 2020;85:140‐144. 31732218 10.1016/j.neurobiolaging.2019.10.001 PMC6917472

[30] Yang Y , Seok MJ , Kim YE , et al. Adeno‐associated virus (AAV) 9‐mediated gene delivery of Nurr1 and Foxa2 ameliorates symptoms and pathologies of Alzheimer disease model mice by suppressing neuro‐inflammation and glial pathology. Mol Psychiatry. 2022;29. doi:10.1038/s41380-022-01693-6 35902630

[31] Yao PL , Parmar VM , Choudhary M , Malek G . NURR1 expression regulates retinal pigment epithelial‐mesenchymal transition and age‐related macular degeneration phenotypes. Proc Natl Acad Sci USA. 2022;119(28):e2202256119. 35867766 10.1073/pnas.2202256119 PMC9282432

[32] Montarolo F , Martire S , Perga S , et al. NURR1 deficiency is associated to ADHD‐like phenotypes in mice. Transl Psychiatry. 2019;9(1):207. 31455763 10.1038/s41398-019-0544-0 PMC6712038

[33] Montarolo F , Martire S , Chiara F , et al. NURR1‐deficient mice have age‐ and sex‐specific behavioral phenotypes. J Neurosci Res. 2022;100(9):1747‐1754. 35593070 10.1002/jnr.25067 PMC9539971

[34] Al‐Nusaif M , Yang Y , Li S , Cheng C , Le W . The role of NURR1 in metabolic abnormalities of Parkinson's disease. Mol Neurodegener. 2022;17(1):46. 35761385 10.1186/s13024-022-00544-w PMC9235236

[35] Eagle AL , Manning CE , Williams ES , et al. Circuit‐specific hippocampal DeltaFosB underlies resilience to stress‐induced social avoidance. Nat Commun. 2020;11(1):4484. 32901027 10.1038/s41467-020-17825-x PMC7479591

[36] Christmann M , Tomicic MT , Aasland D , Berdelle N , Kaina B . Three prime exonuclease I (TREX1) is Fos/AP‐1 regulated by genotoxic stress and protects against ultraviolet light and benzo(a)pyrene‐induced DNA damage. Nucleic Acids Res. 2010;38(19):6418‐6432. 20511593 10.1093/nar/gkq455 PMC2965218

[37] Nestler EJ . ∆FosB: a transcriptional regulator of stress and antidepressant responses. Eur J Pharmacol. 2015;753:66‐72. 25446562 10.1016/j.ejphar.2014.10.034 PMC4380559

[38] Li W , Li S , Shen L , et al. Impairment of dendrodendritic inhibition in the olfactory bulb of APP/PS1 mice. Front Aging Neurosci. 2019;11:2. 30740049 10.3389/fnagi.2019.00002 PMC6357935

